# Theory of Mind and Emotional Functioning in Fibromyalgia Syndrome: An Investigation of the Relationship between Social Cognition and Executive Function

**DOI:** 10.1371/journal.pone.0116542

**Published:** 2015-01-16

**Authors:** Marialaura Di Tella, Lorys Castelli, Fabrizio Colonna, Enrico Fusaro, Riccardo Torta, Rita B. Ardito, Mauro Adenzato

**Affiliations:** 1 Center for Cognitive Science, Department of Psychology, University of Turin, Turin, Italy; 2 Clinical Psychology and Psycho-Oncology Unit, Department of Neuroscience, University of Turin, Turin, Italy; 3 Rheumatology Unit, “Città della Salute e della Scienza” University Hospital of Turin, Turin, Italy; 4 Neuroscience Institute of Turin, Turin, Italy; University of Udine, ITALY

## Abstract

**Background:**

Fibromyalgia (FM) is a syndrome primarily characterised by chronic, widespread musculoskeletal pain. In the aetiology of this syndrome a crucial role is played by complex interactions among biological, genetic, psychological, and socio-cultural factors. Recently, researchers have started to explore emotional functioning in FM, with their attention focused on alexithymia, a personality construct that affects the regulation of a person’s own emotions. On the other hand, the detection and experience of emotional signals from other people have only been sparsely investigated in FM syndrome and no studies have investigated the ability to represent other people’s mental states (i.e. Theory of Mind, ToM) in these patients. Here we present the first study investigating a large set of social-cognitive abilities, and the possible relationships between these abilities and the performance on executive-function tasks, in a homogenous sample of patients with FM.

**Methodology:**

Forty women with FM and forty-one healthy women matched for education and age were involved in the study. Social cognition was assessed with a set of validated experimental tasks. Measures of executive function were used to test the correlations between this dimension and the social-cognitive profile of patients with FM. Relationships between social-cognitive abilities and demographic, clinical and psychological variables were also investigated.

**Principal Findings:**

Patients with FM have impairments both in the regulation of their own affect and in the recognition of other’s emotions, as well as in representing other people’s mental states. No significant correlations were found between social cognition tasks and the subcomponents of the executive function that were analysed.

**Conclusions:**

The results show the presence of several impairments in social cognition skills in patients with FM, which are largely independent of both executive function deficits and symptoms of psychological distress. The impairments reported highlight the importance of adequately assessing ToM and emotional functioning in clinical practice.

## Introduction

Fibromyalgia (FM) is a syndrome primarily characterised by chronic, widespread musculoskeletal pain [1–2]. Its prevalence is estimated to be 3–6% of the world population [3], and it occurs predominantly in women, with a female to male ratio of 10:1 [4–6]. FM symptoms are not restricted to pain, but often include a heterogeneous group of other conditions, such as hyperalgesia and/or allodynia, physical and mental fatigue, disrupted or non-restorative sleep, headache, irritable bowel, psychiatric disorders, cognitive impairment, and other functional complaints [1, 7].

The aetiology of this syndrome is not completely understood, but a crucial role seems to be played by complex interactions among biological, genetic, psychological, and socio-cultural factors, such as medical illness, neuroendocrine disturbances, stress, and psychiatric disorders. In particular, high levels of stress and psychiatric symptoms may negatively influence the perception of disease severity, functional ability, and the threshold and tolerance for pain [8–11]. Some authors have indeed suggested that the development of FM might stem from stress-induced disruption of the hypothalamic-pituitary-adrenal (HPA) axis [12]. Exposure to prolonged stressful conditions can alter the function of the HPA axis, with a consequent increase in the production of corticotropin-releasing factor and potentially amplified pain perception. For this reason, FM is often defined as a *central sensitisation syndrome*, caused by increased sensitivity of the central nervous system to pain signals [13].

Among psychological factors, the high prevalence of depression (20–80%) and anxiety disorders (13–64%) has been widely reported [14–15]. Only recently, researchers have also started to explore emotional functioning in FM syndrome, with their attention focused on alexithymia, a multifaceted personality construct that affects the regulation of a person’s own emotions [16–20]. Alexithymia is characterised by difficulty in identifying and describing subjective feelings, difficulty in distinguishing between feelings and bodily sensations of emotional arousal, restricted imagination processes, and a stimulus-bound, externally oriented cognitive style [21–22]. Most of these studies have reported high levels of alexithymia in patients with FM, suggesting the presence of a deficit in emotional self-awareness.

On the other hand, the detection and experience of emotional signals from other people have only been sparsely investigated in FM syndrome. A link between alexithymic traits and deficits in the processing of other people’s emotions has been highlighted in both healthy individuals and specific clinical populations, e.g. affective disorders, eating disorders, borderline and psychopathic personality disorders, schizophrenia, somatoform disorders [22–26]. To the best of our knowledge, only one study to date has examined the ability to identify other people’s emotions in FM syndrome [27]. The results of this study highlighted the fact that patients with FM had reduced performance in a face-recognition task, with a higher percentage of misclassifications of emotional expressions compared with healthy controls. In addition, pain intensity, alexithymia, depression, and anxiety were inversely related to recognition performance, while psychiatric co-morbidity and medication had no impact on performance.

The ability to decipher information about the intentions and affective states of social partners is crucial for the implementation of appropriate behaviour during social interactions. This complex process is part of the so-called *social cognition domain*, defined as the ability to construct mental representations of the relations that exist between oneself and others and to flexibly use these representations to function effectively in one’s social environment [28–29]. Examples of social cognition abilities are the capacity to represent other people’s intentions and beliefs (i.e. Theory of Mind, ToM) [30–31], and the ability to share and recognise the emotions and sensations of others [32–33].

From a neurological point of view, ToM and emotional processing abilities are associated with overlapping but distinct brain networks [34–36]. Common areas of activation are the prefrontal cortex, the superior temporal sulcus, and the temporo-parietal junctions. These areas form the basis for making inferences about mental states [37–41]. However, these areas are not sufficient for the evaluation of one’s own and other’s emotions, and it is necessary to recruit the additional involvement of emotional networks, in particular those of the amygdala [34, 36, 42–43]. In fact, while the amygdala is not involved in ToM processes per se [44], its role is crucial in processing basic and social emotions, related both to the self and to others [36, 45–48].

The social cognition domain includes a series of different abilities, which gradually evolve throughout the lifetime. An open issue concerns the relationship between these capacities, particularly ToM processes, and the higher-level cognitive skills known as executive functions (EF). Growing numbers of studies are trying to address this relationship in patients with different psychiatric and neurological conditions [49–57].

EF refers to all of the skills that people use to control and coordinate their cognitive abilities and behavior. These are essential for independent everyday functioning in life, and for the establishment of adaptive social relations [58–59]. In the last decade, evidence for the multifaceted nature of EF has replaced the idea of a unitary function [60–63]. Among the several classifications that have been proposed to distinguish EF subcomponents, the model of Miyake et al. [63] identified three separate types of operations: Updating, Shifting, and Inhibition. *Updating* is related to working memory and requires monitoring and coding information as well as replacing old non-relevant information with new relevant information. *Shifting* implies the ability to shift attention between different sub-tasks or different elements of the same task. *Inhibition* is concerned with the individual’s ability to withhold dominant, automatic or prepotent responses when they are inappropriate, and is considered to be a key component in planning abilities. Fisk and Sharp [64] later added a fourth subcomponent, and revised the model of Miyake and colleagues. The factor, called *Access*, refers to the processes involved in verbal fluency tasks, which are believed to mediate access to representations in long-term memory.

Currently, there are two opposing views about the relationship between ToM abilities and EF. Some authors believe that ToM is a circumscribed cognitive process, independent of general intellectual functioning and other cognitive domains, included EF [65]. Basing their ideas on theoretical and experimental data, others have suggested instead that lower-level perceptual abilities (e.g. detection of gaze direction and voice recognition) that are required for an appropriate implementation of ToM skills, may be related to specific and circumscribed cognitive domains, while higher-order ToM processes, involving interpreting and associating information as well as hypothesising, would be the result of a more general ability regarding metarepresentation and EF [66].

The present study is based on both clinical evidence, which has highlighted high levels of emotional distress (depression, anxiety, and in particular, alexithymia) in patients with FM, and neuroimaging and neuropsychological data that has reported functional and structural alterations in brain areas crucial for ToM and emotional processing abilities (i.e. the prefrontal cortex and amygdala) in these patients [67–70], as well as cognitive deficits in the EF domain [69–72]. On these bases, the present study aimed to address two main objectives. The first goal was to evaluate the social-cognitive profile of patients with FM, and analyse ToM and emotional processing abilities. In particular, four different areas of the social cognition domain were examined: (1) regulation of one’s own emotions; (2) empathic capacities; (3) recognition of other’s emotions; (4) representation of other people’s affective mental states (i.e., affective ToM). The second goal was to explore the possible relationships between the performance on executive-function tasks and performance on social-cognition tasks in patients with FM. Furthermore, relationships between social-cognitive abilities and demographic, clinical and psychological variables were also investigated for explorative purposes.

## Materials and Methods

### Ethics Statement

The study was approved by the San Giovanni Battista University Hospital’s ethics committee and was conducted in accordance with the Declaration of Helsinki. All the participants gave their written informed consent to participate in the study.

### Participants and procedure

Forty female participants with FM (51.75 ± 7.76 years of age) were consecutively recruited from the Fibromyalgia Integrated Outpatient Unit (FIOU), a multidisciplinary unit based on the collaboration between rheumatologists, psychologists, and psychiatrists at the San Giovanni Battista University Hospital of Turin. All patients had a main diagnosis of fibromyalgia, made by rheumatologists who are experts in the field. In addition, a psychiatric interview based on DSM IV-TR axis II criteria (Diagnostic and Statistical Manual of Mental Disorders, 4th edition, Text Revision) [75] was performed by an expert psychiatrist, in order to exclude FM patients with personality disorders. Exclusion criteria were as follows: less than 18 years old, low education level (<5 years), and the presence or history of a neurological or a severe psychiatric disorder. Forty-one healthy women (51.83 ± 7.78 years of age) were recruited to the HC group. Exclusion criteria for the HC group were the presence of rheumatic diseases or chronic pain, as well as the presence or history of a neurological or psychiatric disorder.

### Pain evaluation

As an index of pain intensity, the item “Pain” of the Italian version of the Fibromyalgia Impact Questionnaire (FIQ) [76–77] was used to assess the average intensity of pain in the previous week on a scale ranging between 0 and 10.

### Psychological assessment

The presence of symptoms of depression and anxiety was assessed using the Italian version of the Hospital Anxiety and Depression Scale (HADS) [78–79]. It consists of 14 items on a 0 to 3 range, and is divided into two subscales, one for depression (HADS-D) and one for anxiety (HADS-A). Each subscale score ranges from 0 to 21 and a score of 8 (cut-off) or more suggests a level of depression/anxiety symptoms that is clinically relevant [80].

### Neuropsychological assessment

Neuropsychological assessment was performed, using tests for short-term memory (Digit Span-Forward—DS F) [81], learning (Rey auditory-verbal learning test—AVLT) [82], and attention (Trail-making test—TMT—A-B) [83]. For the investigation of executive functions, four different tests were used, specific for each one of the four subcomponents into which EF has been divided according to the models of Miyake et al. [63] and Fisk and Sharp [64]. Specifically, the Digit Span-Backward (DS B) [81] was employed for evaluating the Updating component, the TMT B [83] for Shifting, the Tower of London (ToL) [84–85] for Inhibition, and the verbal fluency (FAS) [82, 86] for Access.

### Social cognition assessment

**Twenty-Item Toronto Alexithymia Scale (TAS-20)**. Alexithymia was assessed using the Italian version of the Toronto Alexithymia Scale (TAS-20) [87–88]. Subjects were asked to indicate the extent to which they agreed or disagreed with each statement on a five-point Likert scale. The results provide a TAS-20 total score, and three subscale scores that measure different aspects of alexithymia: *difficulty identifying feelings* (Factor 1), which measures the inability to distinguish specific emotions and between emotions and the bodily sensations of emotional arousal; *difficulty describing feelings* (Factor 2), which assesses the inability to verbalise one’s emotions to other people; and *externally-oriented thinking* (Factor 3), which evaluates the tendency of individuals to focus their attention externally and not on the inner emotional experience [88–89]. The TAS-20 cut-off scores are as follows: ≤51 no alexithymia, 52–60 borderline alexithymia, ≥61 alexithymia. This scale has shown good internal consistency and test-retest reliability, as well as convergent, discriminant and concurrent validity [22], and it is currently one of the most utilised instruments in studies of alexithymia and emotion.

**Empathy Quotient (EQ)**. The EQ is a validated self-report questionnaire, employed to assess the capacity to empathise with another, i.e. to recognise another’s affective state and to respond to this with an appropriate emotion [90–91]. The EQ comprises 60 items, broken down into two types: 40 items assessing empathy and 20 filler/control items, included to distract the participant from a relentless focus on empathy. For each empathy item, a person can score 2, 1, or 0, so the EQ has a maximum score of 80 (higher scores indicate greater empathy). The EQ is able to detect considerable individual, gender, and group differences, in both general-population and clinical samples.

**Ekman 60 Faces**. The Italian version of this test was used to assess the recognition of facial expressions pertaining to basic emotions [92]. The Ekman 60 Faces Test uses photographs of the faces of 10 actors (six female and four male) selected from the Ekman and Friesen [93] series. Each actor displays one of the six basic emotions investigated (happiness, sadness, disgust, fear, surprise, and anger). The subject is required to respond verbally, deciding which of the six labels for basic emotions that are placed below each photograph can best describe the facial expression shown. The maximum test score (indicating best performance) is 60 for all six emotions and 10 for each basic emotion.

**Reading the Mind in the Eyes Test (RME)**. The RME was employed to assess the ability to represent other people’s affective mental states [94]. In the test, the experimenter presents a set of 36 photographs of the eye region of various human faces. Participants are required to choose among four words that are printed on the page that the picture appears on, using the criterion of which word best describes the mental state of the person depicted in the photograph. Participants have unlimited time to decide, and a glossary is provided. Participants have to put themselves into the mind of another person and recognise his or her complex mental state. In the gender-recognition control task, participants are asked to judge the gender of the person in each of the 36 photographs. For both the experimental (mental state attribution) and control (gender attribution) conditions, the maximum score indicating the best performance is 36.

### Statistical analyses

All the statistical analyses were conducted using IBM SPSS Statistics, version 20.0. Normal distribution was assessed using indices of asymmetry and kurtosis. Non-parametric equivalent tests were performed on data that violated this assumption. For normally distributed variables, independent *t*-tests were used. In order to evaluate the possible relationships between variables, Spearman or Pearson correlations were computed, as appropriate.

## Results

### Demographic, clinical and psychological data

Data on the demographic and psychological variables are presented in Table 1. The two groups were matched for age and education.

**Table 1:** Demographic, clinical and psychological characteristics of the FM and HC groups.

		**FM Patients** **(N = 40)**	**Healthy Controls** **(N = 41)**	**Test (df)**	***p***
**Age, years**	**Mean rank**	41.29	40.72	Z = -0.109	0.913
**Years of education**	**Mean (SD)**	11.50 (3.33)	12.76 (3.68)	t(79) = -1.610	0.111
**Duration of illness, years**	**Mean (SD)**	6.47 (5.81)	-	-	-
**FIQ-Pain**	**Mean (SD)**	7.00 (2.55)	-	-	-
**HADS Total**	**Mean (SD)**	19.63 (6.57)	11.12 (5.76)	t(79) = 6.205	<0.001
	**n% (≥15)**	33 (82.5%)	11 (26.8%)	*Χ* ^2^(1) = 25.289	<0.001
**HADS D**	**Mean (SD)**	9.58 (3.78)	5.05 (3.13)	t(79) = 5.874	<0.001
	**n% (≥8)**	27 (67.5%)	8 (19.5%)	*Χ* ^2^(1) = 19.000	<0.001
**HADS A**	**Mean (SD)**	9.73 (3.78)	5.85 (3.49)	t(79) = 4.785	<0.001
	**n% (≥8)**	27 (67.5%)	14 (34.1%)	*Χ* ^2^(1) = 9.011	0.003

FM = Fibromyalgia, df = Degrees of freedom, FIQ-Pain = item “Pain” of the Fibromyalgia Impact Questionnaire, HADS = Hospital Anxiety and Depression Scale, HADS-A and HADS-D = Anxiety and Depression subscales of the Hospital Anxiety and Depression Scale, SD = Standard deviation.

For psychological assessment we used data from the HADS total score and the scores for subscales HADS-A and HADS-D. The results showed significantly higher scores in patients with FM both for the total score (*p*< 0.001) and for each of the two subscales evaluating anxiety (*p*< 0.001) and depression (*p*< 0.001). According to the cut-off scores of the HADS, 67.5% (27/40) of the patients with FM showed a clinically relevant level of both anxiety and depression, compared with 34% (14/41) for anxiety (*Χ* ^2^(1) = 9.011, *p*< 0.001) and 19.5% (8/41) for depression (*Χ* ^2^(1) = 19.000, *p*< 0.001) in the HC group.

Concerning the clinical characteristics of the FM group, patients reported 6.47 (± 5.81) years of duration of illness and a high rate of pain intensity (7 ± 2.55 to the item “Pain” of the FIQ).

### Neuropsychological assessment

The comparisons between the neuropsychological scores of the two groups are shown in Table 2. Patients with FM performed worse than the HC group on all the four tasks evaluating EF (*p* values ranging from < 0.001–0.011). Furthermore, a poorer performance in the FM group also emerged in the DS F (*p* = 0.005), in the AVLT-Delayed recall (*p* = 0.006) and in the TMT B-A (*p* = 0.004). No statistically significant differences were found on the other neuropsychological measures.

**Table 2:** Neuropsychological tests scores. Mean (SD) or mean rank, t-test or Mann–Whitney *U* test are listed.

	**FM Patients** **(N = 40)**	**Healthy Controls** **(N = 41)**	**Test (df)**	***p***
DS F	4.93 (1.05)	5.61 (1.07)	t(79) = -2.911	0.005
AVLT	46.95 (9.43)	50.73 (8.72)	t(79) = -1.875	0.064
AVLT D	9.98 (2.90)	11.63 (2.39)	t(79) = -2.818	0.006
TMT A	45.41	36.70	Z = -1.669	0.095
TMT B-A	48.58	33.61	Z = -2.863	0.004
*Executive functions measures*				
FAS	38.45 (11.31)	45.73 (9.07)	t(79) = -3.640	< 0.001
DS B	3.59 (0.94)	4.54 (1.08)	t(78) = -4.299	< 0.001
ToL	26.10 (4.40)	28.78 (4.82)	t(79) = -2.610	0.011
TMT B	48.86	33.33	Z = -2.972	0.003

FM = Fibromyalgia, df = Degrees of freedom, DS F = Digit Span Forward, AVLT = Rey auditory-verbal learning test, AVLT D = Rey auditory-verbal learning test Delayed recall, TMT = Trail Making Test, FAS = verbal fluency, DS B = Digit Span Backward, ToL = Tower of London (total score—range 0–36—given by the sum of the time-based scores of the twelve configurations to solve; the higher the score the higher the performance), SD = Standard deviation.

In order to bring out the individual differences that could be flattened by group analyses, the individual scores were analysed comparing for each test the number of subjects with impaired or borderline performance according to the age- and education-corrected scores (equivalent score ≤1). The results showed that a significantly higher number of patients with FM compared with HC had a deficient performance in the DS F (short-term memory) and B (working memory), and in the Delayed recall task of AVLT (episodic memory) (see Table 3).

**Table 3:** Number and percentage of patients with FM and healthy controls with impaired or borderline performance (E.S. = 0–1) at neuropsychological measures.

	**FM Patients** **(N = 40)**	**Healthy Controls** **(N = 41)**	**Test (df)**	***p***
DS F	17 (42.5%)	9 (22%)	*Χ* ^2^(1) = 3.923	0.048
AVLT	5 (12.5%)	1 (2.4%)	*Χ* ^2^(1) = 2.988	0.084
AVLT D	5 (12.5%)	0 (0%)	*Χ* ^2^(1) = 5.462	0.019
TMT A	0 (0%)	0 (0%)	-	-
TMT B-A	1 (2.5%)	0 (0%)	*Χ* ^2^(1) = 1.038	0.308
*Executive functions measures*				
FAS	2 (5%)	0 (0%)	*Χ* ^2^(1) = 2.102	0.147
DS B	18 (45%)	6 (14.6%)	*Χ* ^2^(1) = 8.954	0.003
ToL	2 (5%)	1 (2.4%)	*Χ* ^2^(1) = 0.372	0.542
TMT B	1 (2.5%)	0 (0%)	*Χ* ^2^(1) = 1.038	0.308

FM = Fibromyalgia, df = Degrees of freedom, DS F = Digit Span Forward, AVLT = Rey auditory-verbal learning test, AVLT D = Rey auditory-verbal learning test Delayed recall, TMT = Trail Making Test, FAS = verbal fluency, DS B = Digit Span Backward, ToL = Tower of London.

### Social cognition tasks

Data from social cognition tasks are reported in Table 4.

**Table 4:** Theory of Mind and emotional functioning measures scores.

	**FM Patients** **(N = 40)**	**Healthy Controls** **(N = 41)**	**Test (df)**	***p***
*Regulation of one’s own emotions (Alexithymia)*				
**TAS-F1**	21.78 (6.77)	14.24 (5.83)	t(79) = 5.368	**<0.001**
**TAS-F2**	15.30 (4.72)	12.61 (4.52)	t(79) = 2.619	**0.011**
**TAS-F3**	18.00 (3.44)	17.59 (4.66)	t(73.641) = 0.456	0.649
**TAS-20 Total**	54.75 (9.93)	44.56 (10.09)	t(79) = 4.579	**<0.001**
*Empathic capacities*				
**EQ**	45.95 (9.00)	48.44 (8.54)	t(79) = -1.277	0.205
*Recognition of other’s emotions*				
**Ekman Anger**	7.98 (1.37)	8.61 (1.50)	t(79) = -2.002	**0.049**
**Ekman Sadness**	7.55 (1.55)	8.00 (1.20)	t(73.540) = -1.456	0.150
**Ekman Fear**	6.15 (2.53)	6.54 (2.64)	t(79) = -0.673	0.503
**Ekman Surprise**	37.43	44.49	Z = -1.502	0.133
**Ekman Disgust**	34.89	46.96	Z = -2.401	**0.016**
**Ekman Happiness**	38.96	42.99	Z = -1.111	0.266
**Ekman Total**	48.78 (4.80)	51.41 (4.23)	t(79) = -2.627	**0.010**
*Representation of other people’s affective mental states (Theory of Mind)*				
**RME Experimental**	24.53 (3.81)	26.80 (3.55)	t(79) = -2.787	**0.007**
**RME Control**	43.38	38.68	Z = -0.973	0.331

FM = Fibromyalgia, df = Degrees of freedom, TAS-20 = Twenty-item Toronto Alexithymia Scale, TAS-F1 = Difficult identifying feelings factor of Toronto Alexithymia Scale, TAS-F2 = difficulty describing feelings factor of Toronto Alexithymia Scale, TAS-F3 = externally-oriented thinking factor of Toronto Alexithymia Scale, EQ = Empathy Quotient, RME = Reading the Mind in the Eyes, SD = Standard deviation.

**Regulation of own emotions**. Concerning alexithymia, statistical analyses revealed the presence of significant differences between FM and HC on the TAS-20 total score (*p*< 0.001), and on the F1 (*p*< 0.001) and F2 subscales (*p* = 0.011); in all these comparisons patients with FM scored higher than HC. According to the TAS-20 cut-off scores, 27.5% (11/40) of the patients with FM were alexithymic and 45% (18/40) were borderline, compared with 7% (3/41) and 19.5% (8/41), respectively, in the HC group.

**Empathic capacity**. No significant difference between the two groups was found in the EQ score.

**Recognition of others emotions**. Concerning the Ekman 60 Faces Test, independent *t*-tests revealed the presence of significant differences between the two groups on total score (*p* = 0.010), and on two of the six emotions investigated by means of the test, i.e. anger (*p* = 0.049) and disgust (*p* = 0.016). Once again, patients with FM showed significantly lower scores, indicating a reduced ability to recognise other people’s emotions, especially anger and disgust.

**Representation of other people’s affective mental states**. No significant difference between FM and HC was found in the control task for RME. In the experimental condition, patients with FM evidenced a significantly lower performance on the mental states attribution task (*p* = 0.007).

### Correlations

The second aim of this study was to investigate the possible relationships between social cognition tasks and EF measures in patients with FM. Moreover, we also evaluated the correlations between social cognition tasks and demographic, clinical and psychological data. To do that, we only considered the variables that showed a significant difference in the comparison between FM and HC, i.e. TAS-20 F1, F2, and total score; Ekman anger, disgust and total score; and RME experimental task.

Correlations between social cognition measures and EF tasks are listed in Table 5. As shown, no significant correlation was found, with the only exception of a low positive correlation between the Ekman total score and the DS B. In addition, we verified the possible relationships between social cognition measures, EF tasks and duration of illness (DI) in FM group and no significant correlation emerged (DI and FAS: *r* = -0.276, *p*: ns; DI and DS B: *r* = -0.132, *p*: ns; DI and ToL: *r* = 0.196, *p*: ns; DI and TMT B: *r_s_* = 0.185, *p*: ns; DI and TAS-20 F1: *r* = 0.319, *p*: ns; DI and TAS-20 F2: *r* = 0.333, *p*: ns; DI and TAS-20 Total: *r* = 0.342, *p*: ns; DI and Ekman Anger: *r* = 0.087, *p*: ns; DI and Ekman Disgust: *r_s_* = -0.175, *p*: ns; DI and Ekman Total: *r* = -0.036, *p*: ns; DI and RME Experimental: *r* = -0.053, *p*: ns).

**Table 5:** Pearson or Spearman correlations in FM group between the four executive function measures and TAS-20, Ekman and RME.

	**FAS**	**DS B**	**ToL**	**TMT B**
TAS-F1	*r* = -0.206 *p* = 0.203	*r* = -0.077 *p* = 0.641	*r* = -0.200 *p* = 0.215	*r_s_* = 0.244 *p* = 0.129
TAS-F2	*r* = -0.078 *p* = 0.631	*r* = 0.198 *p* = 0.226	*r* = 0.165 *p* = 0.309	*r_s_* = -0.151 *p* = 0.351
TAS-20 Total	*r* = -0.183 *p* = 0.258	*r* = 0.113 *p* = 0.495	*r* = 0.061 *p* = 0.708	*r_s_* = 0.019 *p* = 0.906
Ekman Anger	*r* = -0.085 *p* = 0.600	*r* = 0.000 *p* = 1000	*r* = 0.184 *p* = 0.257	*r_s_* = 0.206 *p* = 0.203
Ekman Disgust	*r_s_* = 0.224 *p* = 0.165	*r_s_* = 0.026 *p* = 0.876	*r_s_* = -0.031 *p* = 0.850	*r_s_* = -0.196 *p* = 0.225
Ekman Total	*r* = 0.259 *p* = 0.106	*r* = 0.322 *p* = 0.046	*r* = -0.125 *p* = 0.443	*r_s_* = -0.311 *p* = 0.051
RME Experimental	*r* = 0.299 *p* = 0.061	*r* = 0.232 *p* = 0.156	*r* = -0.112 *p* = 0.492	*r_s_* = -0.121 *p* = 0.457

FAS = verbal fluency, DS B = Digit Span Backward, ToL = Tower of London, TMT = Trail Making Test, TAS-20 = Twenty-item Toronto Alexithymia Scale, TAS-F1 = Difficult identifying feelings factor of Toronto Alexithymia Scale, TAS-F2 = difficulty describing feelings factor of Toronto Alexithymia Scale, RME = Reading the Mind in the Eyes.

Regarding the relationship between social cognition measures and demographic, clinical and psychological variables, no significant correlations were detected between age, HADS-A, HADS-D, FIQ-pain, and the RME experimental or the Ekman anger, disgust and total score; a positive correlation was only found between the RME experimental and the level of education (*r* = 0.359, *p* = 0.023). However, significant correlations were detected between demographic, clinical and psychological variables and the TAS-20 scores. In particular, positive correlations were found between the TAS-20 total score and the HADS-A (*r* = 0.334, *p* = 0.035), the HADS-D (*r* = 0.630, *p* < 0.001), and the FIQ-pain (*r* = 0.518, *p* = 0.001). Likewise, positive correlations were found between the TAS-20 F1 and the HADS-A (*r* = 0.462, *p* = 0.003), the HADS-D (*r* = 0.476, *p* = 0.002) and the FIQ-pain (*r* = 0.442, *p* = 0.004). Finally, the TAS-20 F2 was positively correlated only with the HADS-D (*r* = 0.537, *p* < 0.001) and the FIQ-Pain (*r* = 0.344, *p* = 0.030).

## Discussion

The present study aimed to address two main objectives. Firstly, we evaluated the social-cognitive profile of patients with FM, investigating ToM and emotional processing abilities. Secondly, we analysed the relationship between EF deficits and social cognition tasks in patients with FM. Correlations between demographic, clinical and psychological variables, and measures of social cognition were also evaluated.

The results highlighted a significant difference between patients with FM and the HC group in most of the social cognition tasks employed. In particular, the FM group showed significantly higher levels of alexithymia, especially in the subscales “Difficulty in identifying feelings” and “Difficulty describing feelings” of TAS-20, compared to the control sample. These data are in line with most of the studies that have evaluated the prevalence of alexithymia in patients with FM [16–20].

A similar significant difference was found for the experimental task of the RME, while no significant difference was observed for the control task. Patients with FM experienced specific difficulties in representing other people’s affective mental states that cannot be attributed to a basic sensory deficit. The Ekman 60 Faces Test results also showed the presence of significant differences (lower performance of patients with FM) between the two groups both for the total score and for two of the six emotions investigated by means of the test, i.e. anger and disgust; no significant differences were found for other emotions. These data are in line with the only study that has investigated the ability to recognise another’s facial emotions in patients with FM [27]. As mentioned above, these authors showed that patients with FM had reduced performance in the facial affect recognition task, with a higher percentage of misclassifications of emotional expressions compared with the HC group.

The only social cognition task, in which no differences between the two groups were found, was the EQ. In this case, the FM group didn’t report a lower capacity for empathy compared to the control group.

Concerning the general cognitive profile, neuropsychological assessment revealed the presence of significant differences in most of the measures. In particular, the FM group displayed significantly lower performance on the verbal fluency (FAS), the DS B and F, the AVLT-Delayed recall, and the TMT B and B-A, compared to HC. These data are consistent with previous studies that have reported cognitive deficits in attention, memory, and EF domains in patients with FM [71–74]. In particular, Park et al. [71] found that patients with FM demonstrated lower performance on measures of working memory, free recall, verbal fluency, and verbal knowledge, but showed intact speed of processing, compared with age- and education-matched controls. Significantly, patients with FM in that study performed no differently from controls who were 20 years older on most cognitive tasks, with the exception of speed of processing and vocabulary. Only self-reported pain on the Arthritis Impact Measurement Scales predicted poor cognitive performance in the FM group. Measures of depression, anxiety, and the McGill Pain Questionnaire scores were all unrelated to poor cognitive performance. Verdejo-Garcia et al. [73] observed that in the Wisconsin Card Sorting Test, women with FM showed poorer performance than healthy women on the number of categories and non-perseverative errors, but not on perseverative errors. Patients with FM also exhibited an altered learning curve in the original Iowa Gambling Task (IGT) (where reward is immediate and punishment is delayed), suggesting compromised emotion-based decision-making. This was not the case in the variant IGT (where punishment is immediate but reward is delayed), suggesting hypersensitivity to reward. Self-reported pain intensity and pain interference were significantly associated with task performance. In contrast, cognitive performance was not associated with measures of negative mood (i.e. affective distress) or duration of pharmacological treatment, and was very mildly associated with personality characteristics [73].

Finally, from a psychological standpoint, our group of patients with FM presented with significantly higher levels of depressive and anxiety symptoms (67.5% in both cases) compared with the HC group. These results corroborate, once again, the high prevalence of psychological distress reported in previous studies of patients with FM [14–15, 19].

To the best of our knowledge, this is the first study to investigate social-cognitive abilities in a homogenous sample of patients with FM. The results show that patients with FM have impairments both in the regulation of their own affect and in the recognition of other’s emotions, as well as in representing other people’s affective mental states. There is evidence that appropriate behaviour in social interactions is determined by the ability to decipher information about the intentions and affective states of social partners. Thus, impairments in facial affect recognition and difficulties in accurately inferring other people’s affective mental states may lead to substantial difficulties in interpersonal contacts (e.g. interaction problems with family and friends, or social isolation), which have been already reported in patients with FM [95]. Furthermore, poor psychosocial functioning and unsatisfactory relationships might contribute to the genesis and maintenance of chronic pain [96], intensifying the symptomatology in individuals with FM.

From a neurological standpoint, the brain networks relevant for pain and emotional processing partially overlap. The amygdala plays a crucial role in sharing emotional experiences and in recognising emotions in oneself and others [34, 36]. In particular, this structure is involved in the decoding of emotional expressions, and modulates the activity of the fusiform gyrus, which constitutes the most prominent face-selective area of the brain. Neuroimaging studies have indeed shown sensitivity of the amygdala to the kind and valence of facially expressed emotions [97–98]. The insular cortex may also be relevant in this context. It has been reported, for instance, that impaired disgust recognition is associated with reduced insula activity [99]. In addition to their prominent role in emotional functioning, the amygdala and the insula are integral parts of the neural network underlying pain. Specifically, both structures are involved in transmitting the affective dimension of pain perception [100] and are altered in patients with FM [69, 101]. The hyperactivity of the pain network due to central nervous system sensitisation, may lead to an increased demand on structures such as the amygdala and insula, reducing the available resources for other functions such as emotional processing.

As far as the second goal of this study is concerned, we investigated whether EF measures in the FM group were related to the different social cognition tasks that were used. In addition, we also analysed the possible relationships between demographic, clinical and psychological variables, and measures of social cognition. Concerning EF tasks, no correlations were found between social cognition tasks and each of the four subcomponents of the EF domain that were analysed. The only exception was represented by a low positive correlation between the Ekman total score and the DS B. Regarding the demographic, clinical and psychological variables, correlation analyses showed no relationship between Ekman total score, anger and disgust, RME experimental, on the one hand, and anxiety, depression and pain intensity, on the other hand. Positive correlations were only detected between the latter measures (anxiety, depression, and pain) and the total score and scores for the F1 and F2 subscales of the TAS-20. This result is consistent with previous studies that have investigated the presence of alexithymia in FM patients [18, 20, 102]. In particular, Steinweg et al. [102] found higher levels of alexithymia in FM patients compared with either general medical or rheumatoid arthritis patients. However, they also revealed that alexithymia was strongly associated with moderate-to-severe depression, but no group differences were detected when mood disturbance was controlled for.

Taken together, as far as the first aim of this study is concerned, the results show the presence of several impairments in social cognition skills in patients with FM. As for the second aim, i.e. to explore the possible relationships between the performance on executive-function tasks and the performance on social-cognition tasks in patients with FM, we found that the latter are largely independent of both EF deficits and symptoms of psychological distress. The only exception seems to be represented by alexithymia; in fact, psychological disorders, but not EF deficits, seem to play a role in explaining the high levels of alexithymia found in the FM sample. Concerning other measures of social cognition, no relationship was found with EF deficits or symptoms of psychological distress. In our sample, impairments in ToM and emotional processing ability appeared to be independent of the EF domain.

This study also has some limitations. Firstly, even though we enrolled an adequate number of patients with FM, our study is still limited by a relatively small sample size. Secondly, the self-reported measures we used might have elicited a bias towards social desirability, masking the real profile of some individuals. Thirdly, although in patients with FM there is evidence of structural and functional alterations in brain areas crucial for ToM and emotional processing abilities (i.e. the prefrontal cortex and the amygdala) [67–70], we didn’t directly measure the activity in these brain structures. Future studies should include neuroimaging evaluations and use performance-based instruments for the analysis of both empathic capacity and alexithymia, in addition to traditional self-reported tests.

In spite of these limitations, the findings reported in the present study represent the first contribution towards understanding the complex social-cognitive profile of patients with FM. The impairments reported in tasks that evaluate ToM and emotional processing abilities highlight the importance of adequately assessing these abilities in clinical practice. In this way, it could be possible for clinicians to plan better pharmacological and/or psychological treatment based on each patient’s needs.
